# Associations between Medical Disorders and Racing Outcomes in Poorly Performing Standardbred Trotter Racehorses: A Retrospective Study

**DOI:** 10.3390/ani13162569

**Published:** 2023-08-09

**Authors:** Chiara Maria Lo Feudo, Luca Stucchi, Giovanni Stancari, Bianca Conturba, Chiara Bozzola, Enrica Zucca, Francesco Ferrucci

**Affiliations:** 1 Equine Sports Medicine Laboratory “Franco Tradati”, Department of Veterinary Medicine and Animal Sciences, Università Degli Studi di Milano, 26900 Lodi, Italy; chiara.lofeudo@unimi.it (C.M.L.F.);; 2 Veterinary Teaching Hospital, Department of Veterinary Medicine and Animal Sciences, Università Degli Studi di Milano, 26900 Lodi, Italy

**Keywords:** equine sports medicine, equine exercise physiology, poor performance, racehorses, Standardbred, trot racing, equine asthma, exertional rhabdomyolysis

## Abstract

**Simple Summary:**

Many subclinical medical disorders are considered as possible causes of decreased performance in racehorses. The present study aims to evaluate the associations between different disorders diagnosed in 248 poorly performing Standardbreds and their racing outcomes in the periods of time around hospitalization and their lifetime careers. In particular, the effects of respiratory, cardiovascular, gastric, and muscular conditions on number of starts, wins, placings, and earnings were investigated. Inflammation of the lower airways was associated with worse racing results in the periods before and after hospitalization and with a shorter and less successful career, confirming its role in performance impairment. Other conditions negatively influencing short-term racing outcomes included dynamic obstructions of the upper airway and gastric ulcers, while the role of exertional rhabdomyolysis was not clear. Finally, exercise-induced pulmonary hemorrhage and cardiac arrhythmias did not seem to affect Standardbred trotters’ performance.

**Abstract:**

Poor performance in racehorses is commonly associated with subclinical diseases. This study aims to evaluate the associations between medical disorders and racing results in Standardbred trotters. The clinical records of 248 poorly performing Standardbreds were retrospectively reviewed, and their racing results were extracted from an online database, concerning the periods 3 months before and 6 months after hospitalization and the entire lifetime. Generalized linear models were used to evaluate the effects of different disorders on racing outcomes. Airway neutrophilia was associated with limiting lifetime starts and wins pre- and post-hospitalization, while mastocytosis was associated with less wins in the post-hospitalization period. Therefore, lower airway inflammation showed both short- and long-term impacts on racing performance. Severe upper airway obstructions and gastric ulcers showed associations with less placings in the post-discharge period but no long-term influence on performance. The significance of exertional rhabdomyolysis was indeterminable, yet interference with the number of starts in the post-discharge period was reported and associated with lower total career earnings. Exercise-induced pulmonary hemorrhage and cardiac arrhythmias were not associated with worse racing outcomes: therefore, their role in poor performance remains unclear.

## 1. Introduction

Poor performance is a major issue in racehorses, which can be defined as a multifactorial syndrome of decreased athletic capacity, resulting from poor genetic potential, inadequate management, and/or the presence of subclinical disorders [1]. Although orthopedic problems are considered the most common cause of poor performance in both Thoroughbred and Standardbred racehorses [2,3], a variety of medical conditions are reported to negatively impair their fitness status and racing results. Among these, respiratory disorders affecting the upper and/or the lower airways are the most commonly reported cause of poor performance [2,4,5]: in fact, the respiratory ssystem is considered the major performance-limiting factor, even in healthy racehorses [6]. Dynamic upper airway obstructions (DUAOs) are thought to impair racing performance due to the airflow limitation and the consequently subeffective gas exchanges [6,7,8]. Although contrasting results have been reported by studies investigating the effects of DUAOs on the athletic capacity of horses [9,10,11,12], which seem to depend on the entity of the obstruction [13], most authors agree in reporting a significant improvement in racing results after surgical treatment [14,15,16].

Concerning the lower airways, mild–moderate equine asthma (MEA) and exercise-induced pulmonary hemorrhage (EIPH) are very frequently reported causes of poor performance, with a prevalence of nearly 100% among racehorses [4,12,17,18,19]. In particular, MEA has been defined as non-septic lower airway inflammation, associated with chronic occasional coughing; mucus accumulation in the trachea; and a mild increase in neutrophils, eosinophils, and/or mast cells in the bronchoalveolar lavage fluid (BAL) [20]. Several studies have reported a negative influence of MEA on racing outcomes in Standardbred and Thoroughbred racehorses [4,12,21,22,23] probably due to gas exchange impairment at the capillary–alveolar level [24], while contrasting results have been found on the effects of MEA on athletic capacity [17,24,25,26] probably due to the differences in MEA definition and fitness parameters adopted by different studies. Conversely, although EIPH is universally considered a major cause of poor performance, inconsistent results have been reported by studies evaluating its associations with racing results [12,23,27,28,29] and athletic capacity [9,30,31].

Cardiovascular diseases represent another possible cause of poor performance in racehorses, probably by contributing to reduced cardiac output [32]. While mild valvular regurgitations are considered normal in trained horses and do not affect performance [33,34], it is difficult to predict the influence of dynamic cardiac arrhythmias on athletic performance. In fact, some arrhythmias (i.e., atrial fibrillation) are recognized as being associated with decreased sports performance or exercise intolerance [35,36], while the clinical meaning of premature complexes seems to depend on their number and sequence; indeed, no consensus on when they should be considered clinically significant has been reached [5,32,34]. Among other medical conditions that are suspected to impair racing performance, equine gastric ulcer syndrome (EGUS) is extremely common, but the evidence of its effects on racing results and fitness is still scarce, and the underlying mechanisms are unclear [37,38,39]. Finally, another important cause of poor performance is exertional rhabdomyolysis (ER) [40,41], although only one study has reported its negative impact on the horses’ fitness [12], and its contribution to racing impairment has not been quantified yet.

The reason for the inconsistency of results obtained by different studies may be due to different inclusion criteria, diagnostic techniques, and definitions of poor performance. While objective measures can be obtained by standardized exercise tests, no consensus has been reached on which parameters can properly quantify racing performance and represent a racehorse’s success [42,43,44]. In fact, some measures reflect the longevity of the racing career (i.e., number of races), while others reflect its quality (i.e., wins, placings, and earnings). For this reason, and to allow comparisons with previous publications, it has been recommended that both measures of longevity and quality are collected by authors wishing to objectively define racing performance [44].

The present study aims to evaluate the associations between subclinical diseases diagnosed in poorly performing non-lame Standardbred racehorses and their racing performance. In particular, the effects of medical disorders on racing results will be considered in the periods before and after hospitalization for the identification of their short-term negative effects and the evaluation of the success of the prescribed treatment. Moreover, the long-term impact of medical disorders on the racing career longevity and quality will be investigated.

## 2. Materials and Methods

### 2.1. Population

The population of the present study was retrospectively selected among the clinical patients referred to the Equine Unit of the Veterinary Teaching Hospital, University of Milan (Italy), between 2002 and 2022. Standardbred trotter horses with a history of poor performance that underwent a standardized incremental test on treadmill were included, and their clinical records were reviewed. A total of 248 Standardbred racehorses met the inclusion criteria of the present study, including 87 mares (35.08%), 141 stallions (56.85%), and 20 geldings (8.07%), aged between 2 and 9 years old (median 3, IQR 2–4 years old). Horses were subjected to physical examination, hematological exams, resting electrocardiography (ECG), and incremental exercise test on high-speed treadmill, performed as described elsewhere [12]. Moreover, to identify possible subclinical causes of poor performance, ancillary diagnostic procedures were selected by the Equine Sports Medicine Unit team, in agreement with owners and/or trainers, for every single patient, based on history, clinical findings, and presumably the affected body system. Lame horses were excluded from the present study as only the associations between internal medicine disorders and poor performance were investigated. Similarly, horses with clinically relevant cardiac valvular regurgitations or arrhythmias, which are incompatible with racing activity, and horses with grade IV recurrent laryngeal neuropathy (RLN) were excluded. All described procedures were performed for diagnostic purposes in clinical patients, and written informed consent for the use of clinical data for research purposes was obtained from all owners or holders.

### 2.2. Continuous ECG Recording during Treadmill Test

To evaluate the presence of dynamic cardiac arrhythmias, a continuous ECG was obtained before, during and after the treadmill test by Holter recording (Cardioline^®^ Click Holter, Trento, Italy) in 226/248 horses, as previously described [45]. Arrythmias were considered as relevant if at least two isolated premature complexes (PCs) were detected during peak exercise (from the velocity at a heart rate of 200 bpm to the end of maximal exercise) or if at least five PCs or pairs of paroxysms of PCs were detected during peak exercise or immediately after [5].

### 2.3. Post-Exercise Serum CK Activity

Post-exercise serum CK activity was assessed in 242/248 patients. Six hours after the end of the treadmill test, blood samples were collected in plain tubes from the left jugular vein and immediately centrifugated. Serum was separated and serum CK activity was measured by an automatic kinetic ultraviolet-visible spectrophotometric method, as previously described [46].

### 2.4. High Speed Treadmill Endoscopy (HSTE)

To identify DUAOs, an HSTE was performed in 234/248 patients. After warm-up at walk (1.5 m/s) and trot (6 m/s) for, respectively, 4 and 3 min, the treadmill was stopped, and a videoendoscope (ETM PVG-325, Storz, Tuttlingen, Germany) was passed through the ventral meatum of the left nasal passage and into the nasopharynx, until the larynx was fully visualized; here, the endoscope was fixed in position by the use of Velcro straps. Then, the treadmill was rapidly accelerated up to maximal speed (determined for each horse based on the results of the incremental treadmill test) until the horse was no longer able to maintain the speed of the belt or until the horse reached a distance between 1600 and 2100 m. The endoscopic images were visualized in real time and recorded to allow slow-motion analysis [47]. The presence on any DUAO was identified and classified as mild (medial deviation of the aryepiglottic folds and epiglottic entrapment), severe (dorsal displacement of the soft palate, nasopharyngeal collapse, epiglottic retroversion, and dynamic laryngeal collapse), or multiple, according to a previously reported classification [13].

### 2.5. Post-Exercise Tracheobronchoscopy

At 30 min after the end of the HSTE, a post-exercise tracheobronchoscopy was performed in 232/248 horses to evaluate the presence of blood in the trachea and mainstem bronchi. Horses were restrained in a stock and with a twitch, and a flexible videoendoscope (EC-530WL-P, Fujifilm, Tokyo, Japan) was passed through the left nasal passage, and the upper and lower airways were visualized [31]. Based on the presence and severity of blood accumulation in the trachea and bronchi, a 0–4 endoscopic score for EIPH was assigned [48].

### 2.6. Bronchoalveolar Lavage (BAL) Collection and Cytological Examination

A lower airway endoscopy at rest with BAL collection was performed in 205/248 horses. With this aim, horses were sedated with detomidine hydrochloride (0.01 mg/kg IV), and the endoscopy was performed as described above. Once the tracheal bifurcation was visualized, 60 mL of a 0.5% lidocaine hydrochloride solution was sprayed to inhibit the coughing reflex; then, the endoscope was further advanced into the right bronchial tree until it was wedged firmly within a segmental bronchus. Here, 300 mL of a 0.9% sterile saline was instilled through a sterile catheter inserted into the biopsy channel of the endoscope and immediately re-aspirated. The collected fluid was stored in sterile ethylenediaminetetraacetic acid tubes and processed within 90 min [24]. A total of 300 μL of pooled BAL were cytocentrifugated (Rotofix 32, Hettich Cyto System, Tuttlingen, Germany) at 26 *g* for 5 min, and the slides were air-dried, stained with May-Grünwald Giemsa and Perl’s Prussian blue, and observed under a light microscope at 400× and 1000× for 400-cell leukocyte differential count and total hemosiderin score (THS) calculation [31].

### 2.7. Gastroscopic Examination

A gastroscopic examination was performed in 169/248 horses after at least 8 h of fasting. Horses were contained in a stock and with a twitch and sedated with detomidine hydrochloride (0.01 mg/kg IV). A videogastroscope (PV-G 34-325, Storz, Tuttlingen, Germany), connected to an aspirator pump (208-ACH, Faset, Trezzano sul Naviglio, Italy), was passed through the left nasal passages, nasopharynx, and esophagus until the stomach was visualized. The stomach was then insufflated with air, and the mucosa was rinsed to allow a better observation of the squamous and glandular *mucosae*, the *margo plicatus*, and the pylorus [39]. The squamous mucosa was evaluated for equine squamous gastric disease (ESGD) and graded 0–4, while the glandular mucosa was evaluated for presence or absence of equine glandular gastric disease (EGGD) [49].

### 2.8. Treatment

Once diagnosis was made, the team of the Equine Unit prescribed proper pharmacological treatment and/or management measures based on the most updated guidelines in the literature. As horses were discharged at the end of the diagnostic protocol, and treatments were performed by owners, trainers, or holders in their stables, we were not able to control whether the therapies were administered as recommended. Moreover, a follow-up clinical examination was not performed after treatment.

### 2.9. Racing Data

To evaluate the sport performance of enrolled horses, the data of their racing results were collected from the official online database for Italian horseracing (HiD Ippica, www.hippoweb.it, accessed on 1 March 2023, Italy), including number of starts, wins, and placings (first three positions). In particular, these data were divided into three periods of time: 3 months before hospital admission, representing the poor performance period; 6 months after discharge, representing the post-treatment period; and the whole lifetime. Moreover, lifetime earnings were recorded. The racing results during the whole lifetime were considered only for those horses that, at the moment of the study, had retired from racing (*n* = 241).

### 2.10. Statistical Analysis

Data were collected on an electronic sheet (Microsoft Excel, Redmond, WA, USA) and analyzed by two commercially available statistical softwares (GraphPad Prism 9.5.1 for MacOS, GraphPad Software, San Diego, CA, USA; JASP 0.17.2.1 Intel, University of Amsterdam, Amsterdam, The Netherlands). First, the normality of data was evaluated by a Shapiro–Wilk test, and descriptive statistics were performed. Continuous and ordinal data are reported as mean ± standard deviation if normally distributed and as median and interquartile range (IQR) if not normally distributed, and categorical data are reported as percentages of prevalence on the whole population. For the evaluation of the effects of different disorders on each racing outcome, generalized linear models (GLMs) were designed, with age and sex considered as adjusting variables. Variables were initially screened using univariable models, and those with a *p* value < 0.25 were selected for inclusion in the multivariable models. Variables showing collinearity and covariance with other variables were excluded. For the outcomes of “number of starts”, “number of wins”, and “number of placings”, in each period of time, data were first transformed by applying the equation y = y + *random* to obtain a normal distribution with an SD = 1 using the *Transform* function (GraphPad Prism), and then GLMs with Gaussian distribution were used. Meanwhile, for the outcome of “lifetime earnings”, log-linked GLMs with Gamma distribution were used, as our data naturally followed a Gamma distribution, and the log-link provided the best model fit based on deviance and Pearson goodness-of-fit tests. The models were built using a manual backwards method of elimination of variables based on the likelihood ratio test *p* value, the deviance and Pearson goodness-of-fit tests, and by comparing the Akaike information criteria (AIC). Only variables that remained significant in the multivariable models, improved the model fit, or were confounders were retained in the final models. Model fit improvement was defined as the decrease in the AIC by >10%, and variables were considered as confounders when influencing the estimates of other variables by >10%. Finally, the ratios between number of wins and number of starts, and between number of placings and number of starts, were compared between 3 months before and 6 months after examination by a Wilcoxon test in order to evaluate improvement following our teams’ therapeutic recommendations. Statistical significance was set at *p* < 0.05.

## 3. Results

### 3.1. Racing Results in the Study Population

All horses were in full training upon admission. Their racing results during the pre- and post-hospitalization periods, and during the whole lifetime, are displayed in Table 1.

### 3.2. Diagnosis

Mean or median BAL leukocyte counts, the THS, the EIPH grade, post-exercise serum CK activity, and ESGD scores, and the prevalence of DUAOs, EGGD, and PCs are shown in Table 2.

### 3.3. Disorders vs. Racing Outcomes before Hospitalization

The results of the univariable models for each racing outcome in the 3 months before hospitalization that were initially selected for inclusion in the multivariable models are reported in Table S1 (Supplementary Materials). The final multivariable models designed for each racing outcome before hospitalization are shown in Table 3. The number of starts during the 3 months before admission increased with increasing age (*p* < 0.001). Sex and the THS were included in the model as they improved its fit but were not significantly associated with the number of starts. The number of wins decreased with increasing age (*p* = 0.041) and increasing neutrophils in the BAL (*p* = 0.025), while it increased with the presence of PCs (*p* = 0.014). The number of placings increased with increasing eosinophils in the BAL (*p* = 0.039); however, for this outcome, it was not possible to build a multivariable model as, when adjusting for age and sex, or when including other disorder variables, the model did not fit significantly better than the null hypothesis.

### 3.4. Disorders vs. Racing Outcomes after Hospitalization

The results of the univariable models for each racing outcome in the 6 months after hospitalization that were initially selected for inclusion in the multivariable models are reported in Table S2 (Supplementary Materials). The final multivariable models designed for each racing outcome after hospitalization are shown in Table 4. The number of starts during the 6 months after discharge increased with increasing age (*p* < 0.001) and decreased with an increasing THS (*p* = 0.007). The number of wins increased with increasing age (*p* = 0.003) and decreased with increasing mast cells percentage in the BAL (*p* = 0.027). BAL neutrophil percentage was retained in the final model since it improved model fit, and, although statistical significance was not reached, a negative trend of association with the number of wins was observed (*p* = 0.051). The number of placings increased with increasing age (*p* = 0.017) and decreased with increasing ESGD score (*p* = 0.027) and with the presence of severe DUAOs (*p* = 0.030). The BAL eosinophil percentage was included in the model as it improved its fit, and serum CK activity acted as a confounder, but these variables were not significantly associated with the number of placings.

### 3.5. Disorders vs. Lifetime Racing Outcomes

The results of the univariable models for each racing outcome in the lifetime career that were initially selected for inclusion in the multivariable models are reported in Table S3 (Supplementary Materials). The final multivariable models designed for each racing outcome in lifetime are shown in Table 5. The number of starts in lifetime increased with increasing age (*p* < 0.001) and was higher in geldings (*p* = 0.012) and lower in mares (*p* < 0.001) compared to stallions; moreover, it decreased with increasing neutrophil percentages in the BAL (*p* = 0.009). The number of wins and placings increased with increasing age (*p* < 0.001) and was lower in mares than stallions (*p* < 0.001). No medical disorder significantly influenced the number of wins and placings in lifetime. Total lifetime earnings increased with increasing age (*p* < 0.001) and decreased with increasing serum CK activity (*p* = 0.029). The BAL eosinophil percentage was retained in the final model as it improved its fit.

### 3.6. Racing Outcomes before vs. after Hospitalization

**Figure 1:**
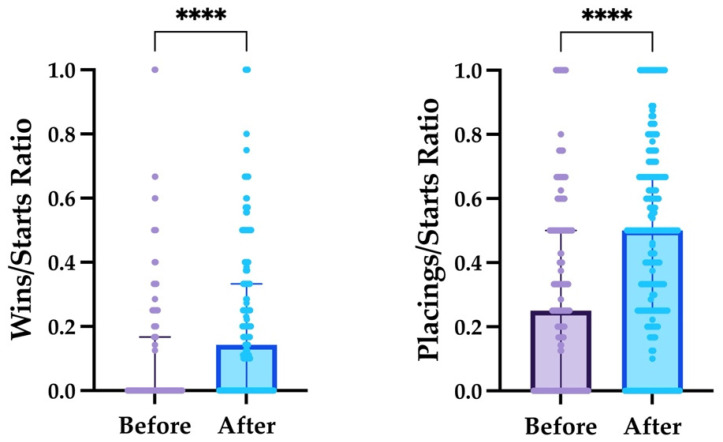
Scatter plots showing the medians (bars) and IQRs (fine lines with serifs) of wins/starts and placings/starts ratios in the study population, before and after hospitalization. Statistical significance is shown as **** (*p* < 0.0001) and was obtained by Wilcoxon test.

The differences between the racing results ratios before and after hospitalization are shown in Figure 1.

## 4. Discussion

Many subclinical medical disorders are considered as possible causes of poor performance in racehorses, but a consensus on their impact on racing results is still far from being reached. The present study aimed to describe the short-term and long-term effects of cardiac arrhythmias, MEA, EIPH, DUAOs, EGUS, and ER on the racing performance of Standardbred trotters. Specifically, we observed both the short-term and long-term negative impacts of MEA on racing career, and a short-term negative effect of severe DUAOs and ESGD on racing outcomes; moreover, ER seemed to contribute to short-term and long-term poor performance, but statistical significance was not achieved. Conversely, cardiac PCs and EIPH did not seem to affect racing performance.

### 4.1. Poor Performance in Racehorses: State of the Art

In the literature, the role in poor performance of respiratory disorders such as MEA, EIPH, and DUAOs has been widely investigated with contrasting results, and only a few studies have attempted to quantify the impact of EGUS, ER, and cardiac arrhythmias on athletic capacity and racing outcomes. The current lack of evidence may be due to the different inclusion criteria of the study populations, ancillary tests used for diagnosis, and selected performance variables. For example, some studies are performed on Standardbred trotters [4,9,13], others on Thoroughbreds [2,8], and others on mixed populations [3,5,10,11]. Lower airway inflammation could be evaluated by BAL [22,24,50] or tracheal wash cytology [4,21,51], DUAOs may be diagnosed by overground [52,53] or high-speed treadmill endoscopy [13,54], and the diagnosis of EIPH may be based on single or multiple tracheobronchoscopies or on BAL cytology [55]. Finally, some studies quantify performance by the measurement of physiological parameters during exercise tests [4,17,25,30], while others quantify performance by collecting racing results [21,22,51]. However, the lack of univocal guidelines on the parameters that should be preferred for racing results evaluation makes it difficult to compare different studies [44]. Therefore, in the present study, we chose to consider a set of variables that could reflect both the longevity and quality of the career. Moreover, to date, most studies investigate the associations between single diseases and performance. However, as poor performance is a complex multifactorial syndrome, rarely caused by a single disorder but, rather, by multiple conditions which could interact between each other [4,5], we chose to evaluate the effects of different medical diseases in this perspective by applying multivariable models.

### 4.2. Mild–Moderate Equine Asthma

Mild–moderate equine asthma has been associated with a more severe hypoxemia during intense exercise [30,56] and a higher peak lactate concentration [56] by some studies, while other authors have reported no association with partial oxygen pressure [9], lactate aerobic–anaerobic threshold [24,30,56], peak lactate concentration [9,24], velocity at 200 bpm, and maximal speed [24,56]. When considering only neutrophilic lower airway inflammation, it has been associated with exercise intolerance [55], reduced speed figure [22], and performance below expectations [4]. Moreover, BAL neutrophilia has been associated with a lower lactate threshold [24,26], velocity at 200 bpm, maximal speed, and higher peak lactate concentration [24]; conversely, other studies failed to detect any relationship between neutrophilic inflammation and physiological fitness variables [17,24]. In one study, an association between BAL mastocytosis and decreased speed figure has been reported too [22]. In our study, neutrophilic lower airway inflammation was associated with a lower number of wins in the periods before hospitalization and with a decreased number of total starts in the lifetime. A trend of association was observed also with less wins in the post-hospitalization period, but this result must be interpreted cautiously due to the lack of statistical significance. These findings confirm the short-term effects of neutrophilic MEA in impairing racing performance but also highlight a negative influence on career longevity. This long-term effect may be explained by the chronic and recurrent nature of MEA. Mastocytic MEA seemed to negatively affect racing performance in the short-term as it was associated with a lower number of wins in the post-hospitalization period; however, unlike neutrophilic inflammation, it did not show any significant effect on lifetime career in the multivariable models. Concerning eosinophilic MEA, to the authors’ knowledge, only one abstract reported, in sport horses, an association between eosinophilic lower airway inflammation and poor performance, with a worse long-term prognosis compared to neutrophilic inflammation [57]. In our study, eosinophilic MEA was associated with a higher number of placings in the pre-hospitalization period, of which clinical meaning remains unexplained; moreover, although statistical significance was not reached, a trend for an association between BAL eosinophilia and lower earnings during the whole racing career was observed. However, these results need to be interpreted cautiously, and further studies should be performed to confirm or confute them.

### 4.3. Exercise-Induced Pulmonary Hemorrhage

Another lower airway condition commonly associated with poor performance is EIPH. Only a few studies reported its relationships with physiological variables: in one of them, EIPH was associated with higher peak lactate concentration [56], while the others did not detect any significant associations with fitness parameters [9,30,31]. Studies investigating the associations between EIPH and racing outcomes are more numerous, and many reported the likelihood of finishing at lower positions in races in EIPH-affected horses [27,29,50,58,59]. Specifically, in a study, grade ≥ 2 EIPH was associated with lower odds of placings and a lower likelihood to fall within the first 90th percentile for earnings, while horses with grade ≥ 1 EIPH finished at a longer distance behind the winner compared to EIPH-negative horses [27]. Other authors reported an association between grade 4 EIPH and a shorter racing career, while no long-term effects on racing performance were detected for grade 1, 2, and 3 EIPH [28]. In other studies, no influence of EIPH on finishing position or racing time was found [23,60,61], and EIPH-affected horses did not have shorter racing careers [62]. Similarly, in our study, we observed no long-term effects of EIPH on career longevity and success; however, it is important to highlight that most included horses were affected by mild grades of EIPH, and this could have prevented us from detecting any possible association with racing outcomes. In the multivariable models, the severity of EIPH seemed to negatively influence the number of starts in the post-discharge period. As in Italy there are no veterinary regulations regarding epistaxis episodes, this finding may be a consequence of the strategic choices of the trainers on how to manage the racing schedules of “bleeder” horses, who may prefer allowing longer periods of rest between races to avoid closely repeated hemorrhage episodes. Furthermore, only in univariable models, EIPH was related with a higher number of starts in the 3 months before examination: in fact, EIPH may be just a consequence of strenuous athletic effort [63]. In particular, there seems to be a threshold below which it is unlikely to have a relevant impact on performance, but EIPH rather consists of a para-physiological result of intense racing [64].

### 4.4. Dynamic upper Airway Obstructions

Among poor respiratory performance causes, DUAOs have also been investigated. Multiple studies have evaluated possible associations between DUAOs and physiological variables; specifically, some have reported an influence of DUAOs on ventilation, arterial blood gases [10,11], and blood lactate concentrations [9], while others did not detect any significant associations [30,65]. In a recent study, it was suggested that DUAOs contributed to impaired ventilation and consequent poor fitness parameters based on the severity of obstruction and the presence of multiple concomitant types of DUAOs [13]. Among the studies based on racing outcomes, one study reported a lower number of starts and earnings in horses affected by grade III RLN, and 2-year-old horses with mild-to-moderate flaccidity of the epiglottis showed lower mean earnings per start compared to control peers [66]. In another study, horses with a moderately to severely flaccid or short epiglottis had less mean total earnings per year and per start [67]. Conversely, other authors found no associations between epiglottic abnormalities and racing performance, while horses with grade III RLN had lower earnings compared to horses with milder forms. In the same study, the dorsal displacement of the soft palate (DDSP) diagnosed as yearlings was positively correlated with racing performance in future years, but the reason for this finding is unknown [68]. Moreover, some studies reported an improvement in racing performance after surgical interventions for RLN or DDSP [14,15,16]. In our study, the presence of severe DUAOs was associated with less placings in the post-hospitalization period, suggesting a negative influence on short-term performance, and a similar trend was observed for multiple concomitant DUAOs. Therefore, as hypothesized by a previous study [13], these findings confirm that DUAOs may contribute to racing performance impairment based on the severity of airflow obstruction. Moreover, it could be difficult to manage those cases in which multiple concomitant forms of DUAO are present as they may require different surgical or medical interventions. However, our study has some limitations, and results should be considered cautiously: first, horses with grade IV RLN were excluded, and second, we are not aware whether horses affected by severe DUAOs underwent surgical treatment after our diagnosis, which could have influenced long-term careers. Hence, we cannot know whether the lack of influence of DUAOs on lifetime racing outcomes is related to the disorder itself or is a consequence of a definitive corrective intervention.

### 4.5. Cardiac Arrhythmias

After respiratory disorders, cardiovascular problems are very common among racehorses, including valvular regurgitations and cardiac arrhythmias. Low-grade murmurs are physiologic in trained horses and do not impair performance, while severe cases may cause not only decreased performance but also safety concerns [32]; therefore, they were not considered in the present study. Among arrhythmias encountered during exercise, supraventricular and ventricular premature complexes have been reported during maximal exercise and immediately after the end of exercise in well-performing racehorses and, when isolated, are considered clinically irrelevant [32,69,70]. To date, evidence-based criteria for the definition of PCs as clinically significant are lacking [70]. In our study, we chose to adopt the classification proposed by Martin and colleagues [5], although this classification is debated. In the study population, we observed that horses with PCs had won more races compared to those without cardiac arrhythmias in the pre-hospitalization period; it could be hypothesized that horses that underwent a more intensive racing program were more prone to develop arrhythmias. However, the clinical significance of this finding is unclear. No other associations were observed, suggesting that the presence of PCs, classified as clinically relevant in the present study, did not negatively affect racing performance nor lifetime career. We cannot exclude that a revised classification of PCs may allow an identification of any effects on performance.

### 4.6. Equine Gastric ulcer Syndrome

Another extremely common disorder in racehorses, which has been associated with poor performance, is EGUS; however, few studies have evaluated objectively this association [49]. Some authors supported this hypothesis by reporting a relationship between the presence of gastric ulcers and racing below trainers’ expectations [36,71,72,73,74]. In a study, an improvement in racing placements was observed after omeprazole treatment in EGUS-affected racehorses [37]. Only two studies reported a significant association between ESGD and worse fitness variables: one performed on naturally affected Standardbred trotters [39] and the other on an experimentally induced model [38]. In the present study, ESGD was associated with a decreased number of placings in the post-hospitalization period, suggesting that EGUS may play a role in poor performance, but its effects are only short-term and evident when the squamous mucosa is affected. However, the lack of association between EGUS and a long-term career may be considered cautiously: indeed, it is possible that EGUS does not effectively impair long-term racing performance or that affected horses may have been treated, and their management changed to prevent relapsing.

### 4.7. Exertional Rhabdomyolysis

Finally, exertional rhabdomyolysis can represent an important issue for poor performance both because of the clinical episodes themselves and the lost training days [5,40,41]. In a study, horses with ER were less likely to race compared to control horses, but, among those who raced, no difference in racing performance was observed [75]; similarly, in another study, ER was not associated with Timeform rating [76]. Other authors found that Standardbreds suffering from ER were faster and had higher winning and placing percentages compared to control horses: this result is surprising, but authors have hypothesized that this may be a consequence of the breeding selection, which may maintain hereditary ER in good performers [77]. In our study, post-exercise serum CK activity was associated with lower lifetime earnings, supporting the hypothesis that ER may negatively impact the racing career probably due to its recurrent nature. 

### 4.8. Study Limitations

In order to confirm the presence of poor performance in the study population at the time of examination, we compared the wins/starts and placings/starts ratios between the periods before and after hospitalization. A significant increase in both ratios was observed after discharge, suggesting that horses were actually performing under their standard capacity at the time of examination and that the prescribed therapy was effective in improving racing outcomes. However, we could not control whether the treatments were accomplished by trainers or owners, and no follow-up examination was performed to verify healing or the improvement of the diagnosed disorders, which represents a limitation of the present study. It could have been interesting to compare racing outcomes before and after examination, divided on the basis of the diagnosis; however, most horses were affected by multiple concomitant disorders, and more than one treatment was prescribed accordingly. Indeed, as poor performance is a multifactorial syndrome [4,5], its therapeutic approach often includes multiple management and pharmacological measures.

Another limitation of our study resides in its retrospective nature; in fact, horses have been included over 20 years of time, during which the clinical approach and equipment availability has slightly varied. For the same reason, data on the total earnings should be considered cautiously, as they are influenced by the quality of the race and the prizemoney available in different racing jurisdictions during a long period of time [64]. Indeed, during the last years, the horseracing industry has undergone a major crisis, with consequent decrease in money invested in racing prizes. Moreover, as only poorly performing horses were included, no data on a group of good performers was obtained, not allowing comparison with a control group. Finally, it should always be taken in mind that poor performance can have several causes which are not necessarily pathological conditions; for example, inadequate training, shoeing, feeding management [78], horses’ mental wellbeing [79], and the individual genetic potential [80], are all factors that may have a huge impact on racing outcomes. Nevertheless, the authors deem that the large size of the population may compensate for these limitations.

## 5. Conclusions

The present study confirmed the role of MEA in racing performance impairment in Standardbred trotters, with both short-term and long-term effects on their careers. Also, the presence of severe DUAOs and the severity of ESGD were associated with short-term poor performance. Another disorder associated with a less successful lifetime career was ER. Finally, no negative effects of EIPH and cardiac arrhythmias on racing performance were detected.

Our results partially support those of previous studies, while they are in contrast with others. This highlights the need for a consensus on racing performance definitions in order to make studies repeatable and comparable between each other. Future standardized studies should be addressed to confirm or refute our results and those of previous studies.

## Figures and Tables

**Table 1:** Racing results of the study population during the 3 months before admission, 6 months after discharge, and during the whole lifetime. Results are expressed as median (interquartile range).

Variable	3 Months before Admission	6 Months after Discharge	Lifetime
Number of starts	4 (2–5)	4 (2–6)	56 (32–95)
Number of wins	0 (0–1)	1 (0–2)	9 (5–16)
Number of placings	1 (0–2)	2 (1–4)	22 (12–41)
Earnings (EUR)	-	-	63,246 (23,382–127,836)
Earnings/Starts (EUR)	-	-	940.20 (508.20–1892)

**Table 2:** Results of the diagnostic procedures in the study population. Results are expressed as mean ± standard deviation if normally distributed, as median (interquartile range) if not normally distributed, or as percentage on prevalence if categorical variables.

Variable	Mean, Median, or Prevalence
Lower Airway Inflammation	
BAL Macrophages (%)	44.74 ± 8.58
BAL Lymphocytes (%)	36.68 ± 11.89
BAL Neutrophils (%)	8 (5–17)
BAL Mast cells (%)	4 (3–6)
BAL Eosinophils (%)	1 (0–3)
Exercise-induced pulmonary hemorrhage	
Total hemosiderin score	30 (9–62)
Tracheal EIPH grade	1 (0–2)
Dynamic upper airway obstructions	
No DUAO (%)	56.96
Mild DUAO (%)	4.64
Severe DUAO (%)	25.74
Multiple DUAOs (%)	12.66
Rhabdomyolysis	
Serum CK (UI/L)	204 (110–410)
Equine Gastric Ulcer Syndrome	
ESGD score	4 (3–4)
EGGD-negative (%)	41.42
EGGD-positive (%)	58.58
Cardiac arrhythmias	
No significant arrhythmias (%)	81.58
Premature complexes (%)	18.42

BAL = Bronchoalveolar lavage; EIPH = exercise-induced pulmonary hemorrhage; DUAO = dynamic upper airway obstruction; CK = creatine-kinase; ESGD = equine squamous gastric disease; EGGD = equine glandular gastric disease.

**Table 3:** Multivariable Gaussian generalized linear models designed for the outcomes of “number of starts”, “number of wins”, and “number of placings” in the 3 months before hospitalization in a population of 248 Standardbred trotter horses referred for poor performance between 2002 and 2021.

Variable	Estimate	95% Confidence Interval	*p* Value
Number of Starts			
Age	0.441	0.215–0.667	<0.001
Sex—Stallion	ref	ref	ref
Sex—Gelding	1.058	−0.038–2.154	0.060
Sex—Mare	−0.407	−1.041–0.227	0.210
THS	0.003	−0.002–0.009	0.239
Number of Wins			
Age	−0.135	−0.264–−0.007	0.041
BAL Neutrophils	−0.021	−0.039–−0.003	0.025
PC—absence	ref	ref	ref
PC—presence	0.556	0.117–0.995	0.014
Number of Placings			
BAL Eosinophils	0.053	0.003–0.104	0.039

THS = Total hemosiderin score; BAL = bronchoalveolar lavage; PC = premature complexes.

**Table 4:** Multivariable Gaussian generalized linear models designed for the outcomes of “number of starts”, “number of wins”, and “number of placings” in the 6 months after hospitalization in a population of 248 Standardbred trotter horses referred for poor performance between 2002 and 2021.

Variable	Estimate	95% Confidence Interval	*p* Value
Number of Starts			
Age	0.638	0.314–0.961	<0.001
THS	−0.012	−0.020–−0.003	0.007
Number of Wins			
Age	0.220	0.079–0.361	0.003
BAL Neutrophils	−0.022	−0.043–−7.519 × 10^−5^	0.051
BAL Mast Cells	−0.103	−0.192–−0.013	0.027
Number of Placings			
Age	0.316	0.061–0.570	0.017
BAL Eosinophils	−0.061	−0.155–0.034	0.212
DUAO—Absence	ref	ref	ref
DUAO—Mild	0.513	−0.929–1.955	0.487
DUAO—Severe	−0.813	−1.539–−0.086	0.030
DUAO—Multiple	−0.812	−1.815–0.192	0.115
Serum CK	−4.656 × 10^−4^	−0.001–2.747 × 10^−4^	0.220
ESGD	−0.432	−0.811–−0.054	0.027

THS = Total hemosiderin score; BAL = bronchoalveolar lavage; DUAO = dynamic upper airway obstruction; CK = creatine-kinase; ESGD = equine squamous gastric disease.

**Table 5:** Multivariable generalized linear models with Gaussian distribution designed for the outcomes of “number of starts”, “number of wins”, and “number of placings” and with Gamma log-linked distribution for the outcome of “earnings” for a lifetime racing career in a population of 248 Standardbred trotter horses referred for poor performance between 2002 and 2021.

Variable	Estimate	95% Confidence Interval	*p* Value
Number of Starts			
Age	8.699	4.434–12.963	<0.001
Sex—Stallion	ref	ref	ref
Sex—Gelding	27.427	6.239–48.616	0.012
Sex—Mare	−35.549	−47.689–−23.408	<0.001
BAL Neutrophils	−0.839	−1.466–−0.213	0.009
Number of Wins			
Age	2.691	1.933–3.449	<0.001
Sex—Stallion	ref	ref	ref
Sex—Gelding	1.221	−2.496–4.938	0.520
Sex—Mare	−4.079	−6.231–−1.927	<0.001
Number of Placings			
Age	5.198	3.541–6.856	<0.001
Sex—Stallion	ref	ref	ref
Sex—Gelding	4.924	−3.204–13.053	0.236
Sex—Mare	−10.188	−14.894–−5.482	<0.001
Earnings			
Age	0.287	0.150–0.433	< 0.001
BAL Eosinophils	−0.039	−0.083–0.012	0.079
Serum CK	−4.434 × 10^−4^	−8.100×10^−4^–−2.587 × 10^−5^	0.029

BAL = Bronchoalveolar lavage; CK = creatine-kinase.

## Data Availability

The data presented in this study are available upon request from the corresponding authors.

## Supplementary Materials

The following supporting information can be downloaded at: https://www.mdpi.com/article/10.3390/ani13162569/s1, Table S1. Results of the univariable models for the outcomes “number of starts”, “number of wins”, and “number of placings” in the 3 months before hospitalization that were initially selected for inclusion in the multivariable models, in a population of 248 Standardbred trotter horses referred for poor performance between 2002 and 2021. Table S2. Results of the univariable models for the outcomes “number of starts”, “number of wins”, and “number of placings” in the 6 months after hospitalization that were initially selected for inclusion in the multivariable models, in a population of 248 Standardbred trotter horses referred for poor performance between 2002 and 2021. Table S3. Results of the univariable models for the outcomes “number of starts”, “number of wins”, “number of placings” and “earnings” in lifetime that were initially selected for inclusion in the multivariable models, in a population of 248 Standardbred trotter horses referred for poor performance between 2002 and 2021.
