# Supplementary material for: Associations between Medical Disorders and Racing Outcomes in Poorly Performing Standardbred Trotter Racehorses: A Retrospective Study
Source: Animals (Basel). 2023 Aug 9;13(16):2569. doi: 10.3390/ani13162569 (PMC10451774; doi:10.3390/ani13162569)
Supplement: Supplementary file 1 [file animals-13-02569-s001.zip › animals-2430255-supplementary.pdf]

Table S1. Results of the univariable models for the outcomes "number of starts", "number of wins", and "number of placings" in the 3 months before hospitalization that were initially selected for inclusion in the multivariable models, in a population of 248 Standardbred trotter horses referred for poor performance between 2002 and 2021.

Variable	Estimate	95% Confidence Interval	*p* value
		Number of Starts	
BAL Mast Cells	0.09	-0.05 – 0.23	0.200
THS	0.01	ref	ref
No DUAO	ref	0.001 – 0.01	0.026
Mild DUAO	0.17	ref	ref
Severe DUAO	0.15	-1.16 – 1.51	0.800
Multiple DUAOs	-0.54	-0.50 – 0.81	0.647
PC - absence	ref	-1.40 – 0.32	0.219
PC - presence	0.63	ref	ref
		Number of Wins	
BAL Neutrophils	-0.02	-0.04 – -0.01	0.014
THS	-0.003	-0.01 – 0.001	0.110
EGGD - absence	ref	ref	ref
EGGD - presence	-0.23	-0.60 – 0.14	0.220
PC - absence	ref	ref	ref
PC - presence	0.35	-0.04 – 0.75	0.083
		Number of Placings	
BAL Eosinophils	0.05	0.003 – 0.10	0.039
Serum CK	-0.001	-0.001 – 0.001	0.207

BAL = bronchoalveolar lavage, THS = total hemosiderin score, DUAO = dynamic upper airway obstruction, EGGD = equine glandular gastric disease, PC = premature complexes, CK = creatine-kinase.

Table S2. Results of the univariable models for the outcomes "number of starts", "number of wins", and "number of placings" in the 6 months after hospitalization that were initially selected for inclusion in the multivariable models, in a population of 248 Standardbred trotter horses referred for poor performance between 2002 and 2021.

Variable	Estimate	95% Confidence Interval	*p* value
		Number of Starts	
BAL Eosinophils	-0.10	-0.21 – 0.004	0.062
THS	-0.01	-0.02 – 0.001	0.060
Serum CK	-0.001	-0.002 – 0.001	0.053
PC - absence	ref	ref	ref
PC - presence	0.75	-0.36 – 1.85	0.187
		Number of Wins	
BAL Eosinophils	-0.04	-0.09 – 0.01	0.088
BAL Neutrophils	-0.02	-0.04 – 0.01	0.174
BAL Mast Cells	-0.06	-0.15 – 0.03	0.185
		Number of Placings	
BAL Eosinophils	-0.04	-0.09 – 0.01	0.050

BAL Neutrophils	-0.02	-0.04 – 0.01	0.226
THS	-0.06	-0.15 – 0.03	0.236
No DUAO	ref	ref	ref
Mild DUAO	-020	-1.51 – 1.10	0.761
Severe DUAO	-0.31	-0.95 – 0.34	0.353
Multiple DUAOs	-0.50	-1.34 – 0.34	0.244
Serum CK	-0.001	-0.001 – 0.001	0.082
ESGD	-0.10	-0.38 – 0.17	0.028

BAL = bronchoalveolar lavage, THS = total hemosiderin score, CK = creatine-kinase, PC = premature complexes, DUAO = dynamic upper airway obstruction, ESGD = equine squamous gastric disease.

Table S3. Results of the univariable models for the outcomes "number of starts", "number of wins", "number of placings" and "earnings" in lifetime that were initially selected for inclusion in the multivariable models, in a population of 248 Standardbred trotter horses referred for poor performance between 2002 and 2021.

Variable	Estimate	95% Confidence Interval	*p* value
		Number of Starts	
BAL Neutrophils	-0.65	-1.38 – 0.08	0.082
Serum CK	-0.01	-0.03 – -0.001	0.042
		Number of Wins	
BAL Eosinophils	-0.24	-0.55 – 0.08	0.138
BAL Mast Cells	0.43	-0.15 – 1.01	0.148
Serum CK	-0.002	-0.005 – 0.001	0.053
		Number of Placings	
Serum CK	-0.01	-0.01 – 0.001	0.054
		Earnings	
BAL Eosinophils	-0.06	-0.11 – -0.01	0.005
BAL Mast Cells	0.06	-0.02 – 0.14	0.151
THS	0.003	-0.001 – 0.01	0.152
No DUAO	ref	ref	ref
Mild DUAO	-0.32	-1.06 – 0.63	0.460
Severe DUAO	-0.08	-0.48 – 0.34	0.690
Multiple DUAOs	-0.38	-0.88 – 0.19	0.170
Serum CK	-0.001	-0.001 – -0.001	0.028

BAL = bronchoalveolar lavage, CK = creatine-kinase, THS = total hemosiderin score, DUAO = dynamic upper airway obstruction.
